# Migration of a Nelaton Catheter Into the Pulmonary Artery During Spinal Surgery Requiring Surgical Removal: A Regrettable Case

**DOI:** 10.7759/cureus.88058

**Published:** 2025-07-16

**Authors:** Eitaro Okumura, Ryo Hashimoto, Hiroki Eguchi, Motoo Kubota

**Affiliations:** 1 Spinal Surgery, Kameda Medical Center, Chiba, JPN

**Keywords:** common iliac vein injury, intravascular foreign body, major complications, pulmonary artery embolism, spinal fusion surgery

## Abstract

Perioperative complications in spinal surgery include dural injury, postoperative epidural hematoma, and surgical site infections. More severe complications involve paralysis, sensory deficits due to nerve injury, and bladder or bowel dysfunction. During fusion surgery, attention must also be paid to risks such as guide wire breakage, cage migration or dislodgement, and major vascular injury during intervertebral disc gauge placement. A common empirical practice during bone drilling with a high-speed drill involves placing a 15 mm segment of a Nelaton catheter over the tip of the suction tube to prevent damage to the tissue. This is referred to as a "Nelaton cover." We report a regrettable case in which a Nelaton cover became dislodged during lumbar disc curettage, subsequently migrating into the common iliac vein and leading to pulmonary artery embolism, which required surgical removal. The patient was a 71-year-old man with independent activities of daily living and a history of diabetes mellitus and lumbar disc herniation (L5/S1). He presented with lower back pain, bilateral lower extremity pain and numbness, and intermittent claudication that had persisted for one year. At presentation, there was no significant muscle weakness, but he experienced pain and numbness extending from the left buttock to the lateral aspect of the left lower leg. Lumbar MRI showed left L5 foraminal stenosis, and posterior lumbar decompression and fusion at L5/S1 was planned. During surgery, following L5 laminectomy, active bleeding was noted within the disc space during L5/S1 disc curettage. A venous injury on the ventral side of the disc was suspected. Hemostasis was attempted using a suction tube fitted with a Nelaton cover. However, after completing the hemostatic maneuver, the Nelaton cover was found to be missing and not visible in the surgical field. The operation proceeded, with disc cage and pedicle screw placement completed as planned. Postoperative lumbar X-ray revealed the Nelaton cover had likely migrated into the inferior vena cava at the L3/4 level. A postoperative CT scan confirmed its presence in the left pulmonary artery. Thoracic surgeons performed surgical removal. Fortunately, the patient showed no decline in pulmonary function, experienced improvement in bilateral leg numbness, and was discharged ambulatory with a modified Rankin Scale score of 1. In this case, bleeding during disc curettage was likely due to common iliac vein injury. During compression hemostasis using the suction tube with a Nelaton cover, the cover became detached and entered the venous circulation, ultimately resulting in pulmonary embolism. This case highlights the need for caution when managing bleeding near major vessels during spinal surgery and underscores the risk of using improvised tools such as Nelaton covers for hemostasis.

## Introduction

Iatrogenic vascular injury during thoracic and lumbar spinal surgery, though rare, is a potentially life-threatening complication with a reported incidence of 0.01% to 1% [1]. In a literature review of 56 studies on vascular complications in thoracolumbar procedures, Lucifero et al. found that, in posterior approaches, the most frequently affected vessels were the common iliac artery and vein, followed by the aorta and lumbar arteries [1]. These injuries typically occur intraoperatively as a result of direct iatrogenic trauma. Akhaddar et al. reported that the average time from vascular injury to the onset of symptoms is approximately 7.3 hours, with deep vein thrombosis and pulmonary embolism being the most commonly associated complications [2]. During lumbar surgery, high-speed drills are commonly used for bone removal, while suction tubes help clear bone fragments from the operative field. To prevent damage to the suction tube tip caused by contact with the high-speed drill, there is an empirical practice of attaching a short segment, approximately 15 mm, of a Nelaton catheter to the tip of the suction tube. This segment, known as a "Nelaton cover," is cut from a flexible catheter typically used in urological procedures such as urethral catheterization, urinary drainage, and bladder irrigation. Before surgery, the suction tube with the Nelaton cover attached is sterilized, and proper attachment is confirmed by the scrub nurse and again by the surgeon before use. Despite these precautions, we report a regrettable case in which, during L5/S1 lumbar disc curettage, a common iliac vein injury occurred, and the Nelaton cover at the tip of the suction tube became dislodged. The detached cover migrated into the common iliac vein and ultimately caused a pulmonary artery embolism.

## Case presentation

**Figure 1:**
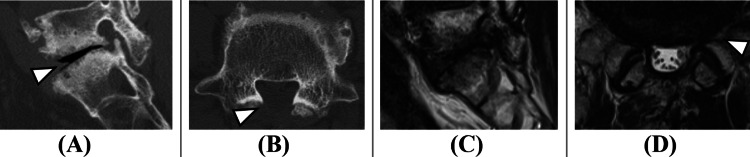
Preoperative images. (A) Sagittal and (B) axial lumbar CT view. (C) Sagittal and (D) axial lumbar MRI view. Following prior lumbar disc herniation surgery, partial resection of the L5 lamina is visible (white arrowhead, B). Disc space collapse, suggestive of segmental instability, is noted (white arrowhead, A). Left L5 foraminal stenosis is also evident (white arrowhead, D), indicating likely compression of the left L5 nerve root and resulting in sciatica symptoms.

The patient was a 71-year-old man with independent activities of daily living and a medical history of diabetes mellitus and lumbar disc herniation at the L5/S1 level. Two years earlier, he had undergone lumbar disc herniation surgery, which included partial resection of the L5 lamina. He presented with persistent symptoms of lower back pain, bilateral lower extremity pain and numbness, and intermittent claudication that had continued for one year without improvement, prompting an outpatient consultation. On examination, there was no obvious muscle weakness in the lower extremities; however, pain and numbness were noted from the left buttock down to the lateral aspect of the left lower leg. Lumbar MRI revealed left L5 foraminal stenosis (Figure 1).

**Figure 2:**
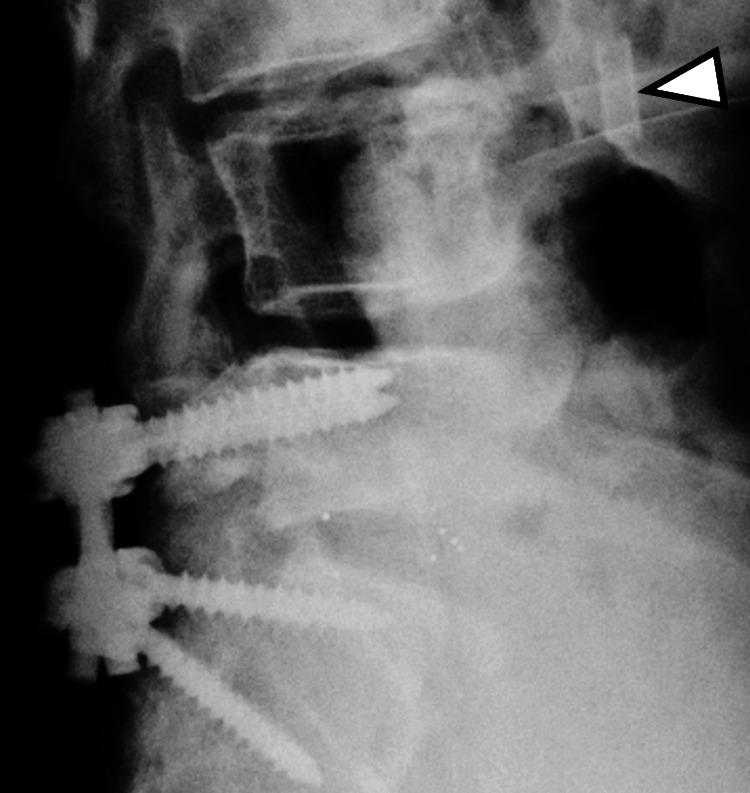
Immediate postoperative lumbar X-ray. A structure suggestive of the Nelaton cover is seen near the superior margin of the L4 vertebral body (white arrowhead).

A left L5 nerve root block was performed, which successfully relieved the patient's pain and confirmed the lesion as the source of symptoms. For definitive treatment, posterior lumbar decompression and fusion surgery at L5/S1 was planned. During surgery, following completion of the L5 laminectomy, active bleeding was observed within the disc space during L5/S1 disc curettage. A venous injury on the ventral side of the disc was suspected, and compression hemostasis was attempted using a suction tube. A Nelaton cover had been attached to the tip of the suction tube; however, after the hemostatic procedure, it was discovered that the Nelaton cover had become detached. Although it was not visible in the surgical field, it was presumed to have moved outside the operative area during saline irrigation or curettage. The scrub nurse and circulating nurse were instructed to search for the missing cover, while the surgeon proceeded with the operation. Consequently, intraoperative imaging was not performed immediately after the Nelaton cover became unaccounted for. After disc cage insertion and pedicle screw placement, the posterior lumbar decompression and fusion surgery were completed. Immediately following wound closure, a lumbar X-ray was obtained. The postoperative image suggested that the Nelaton cover had migrated into the inferior vena cava at the L3/4 level (Figure 2).

**Figure 3:**
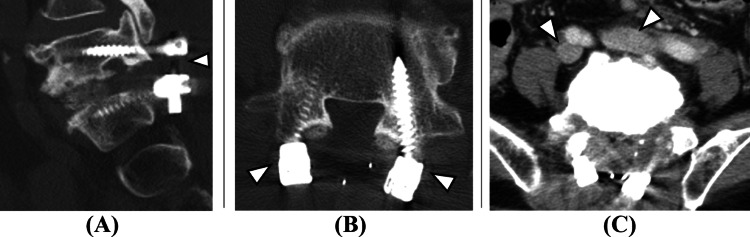
Immediate postoperative lumbar CT. (A) Sagittal, (B) axial, and (C) contrast-enhanced axial view. L5/S1 is stabilized with pedicle screws (white arrowheads, A and B). In the contrast-enhanced axial image at the L5/S1 disc level, the bilateral common iliac veins are seen adjacent to the ventral aspect of the disc. No significant hematoma formation is noted.

**Figure 4:**
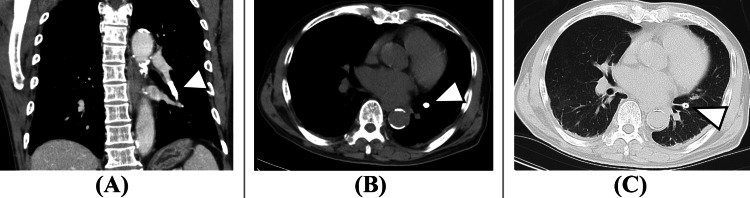
Immediate postoperative chest CT. (A) Contrast-enhanced coronal view. (B) Axial view (soft tissue window). (C) Axial view (lung window). A foreign body consistent with Nelaton cover embolism is visible in the left pulmonary artery (white arrowheads, A-C).

A postoperative CT scan was promptly performed. Initially, no Nelaton cover was detected in the inferior vena cava. However, upon extending the imaging range to include the chest, the Nelaton cover was identified within the left pulmonary artery (Figures 3, 4).

**Figure 5:**
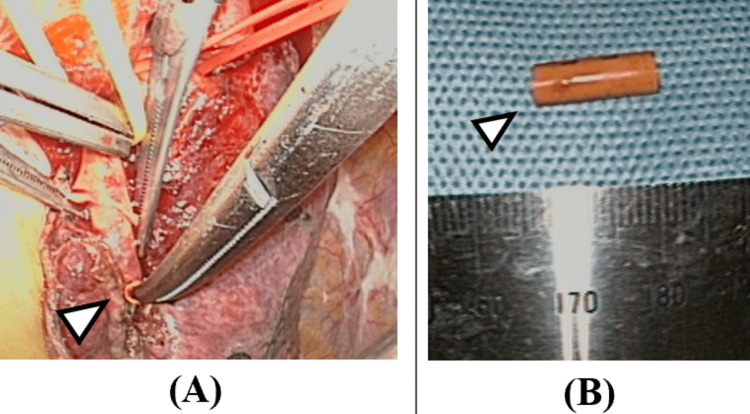
Intraoperative images from thoracic surgery. After clamping the target pulmonary artery, the Nelaton cover was successfully identified and removed (white arrowhead, A). Approximately 12 mm segment of the Nelaton cover was removed in one piece (white arrowhead, B).

As the patient's vital signs, including respiratory status, remained stable, surgical removal of the Nelaton cover was performed by thoracic surgeons on postoperative day 2 (Figure 5). Fortunately, no postoperative decline in pulmonary function was observed. The patient’s sciatica and intermittent claudication improved, and he was discharged ambulatory with a modified Rankin Scale score of 1.

## Discussion

Since the aorta and inferior vena cava bifurcate at the level of L4, spinal instrumentation or surgical manipulation at the L5/S1 level carries a risk of serious vascular injury, particularly to the common iliac artery and vein [3,4]. In fact, venous injuries during transforaminal lumbar interbody fusion (TLIF) have been reported. Ariyoshi et al. described a case of inferior vena cava injury during the removal of a disc cage that had migrated ventrally [5]. Similarly, Murase et al. reported that anterior dislodgement of disc cages, although rare (0.26% incidence), can result in vascular compression. They emphasized the need for cage removal when a major vessel is in contact or compressed to avoid delayed vascular injury [6]. Vascular injury not only poses a risk of hemorrhage but also of thromboembolic events. Wang et al. reported a case in which inferior vena cava injury during percutaneous vertebroplasty led to deep vein thrombosis and subsequent pulmonary thromboembolism [7]. In our case, the disc curettage was initiated from a more lateral approach than usual, and the forceps were inserted more deeply, likely causing a vascular laceration. At the L5/S1 level, CT imaging demonstrates that the common iliac vessels lie in close proximity to the anterior vertebral body. Although active bleeding was observed intraoperatively, the fact that hemostasis was achieved relatively quickly with direct compression suggests a venous rather than arterial injury. Depending on the degree of vascular laceration, laparotomy or endovascular treatment for vascular repair may be necessary [8]. Given that the Nelaton cover migrated intact into the vascular system, a large-caliber vessel, such as the common iliac vein, was most likely involved. The cover likely entered the common iliac vein, traveled through the inferior vena cava, and ultimately embolized in the left pulmonary artery, requiring surgical removal. Fortunately, the Nelaton cover was removed in one piece without fragmentation, and the patient experienced no complications such as deep vein thrombosis or respiratory impairment. Preventive strategies should be considered in light of this case. Preoperative planning should include estimation of safe insertion depths for instruments during disc curettage to avoid breaching the anterior longitudinal ligament. If fat or mucosal tissue is found on forceps tips, this may indicate penetration into the retroperitoneum or vessel wall, and even in the absence of active bleeding, vascular injury must be strongly suspected [9]. Additionally, the routine use of suction tubes with Nelaton covers for hemostasis, outside their intended use in protecting suction tips during high-speed drilling, should be reconsidered. As demonstrated in this case, such use can lead to unforeseen and serious complications. Currently, there are no reports evaluating the frequency or safety of using Nelaton covers in spinal surgery, and no standardized method for their attachment exists. Further systematic research is necessary to assess their utility and safety.

## Conclusions

We report a regrettable case in which a common iliac vein injury occurred during disc curettage. Compression hemostasis was attempted using a suction tube with a Nelaton cover, but the cover became dislodged and migrated into the common iliac vein, ultimately resulting in pulmonary artery embolism. This case highlights the importance of recognizing that iatrogenic vascular injury, though rare, can occur during lumbar spine surgery. When such an injury is suspected or identified, prompt multidisciplinary collaboration and rapid intervention are essential to minimize complications and ensure patient safety.
